# Remarkable Case of Foreign Bodies in the Stomach and Duodenum, Complete Obstruction of the Bowel and Mechanical Displacement of Organs

**Published:** 1854-02

**Authors:** John Marshall


*Remarkable case of Foreign Bodies in the Stomach and Duodenum, Complete Obstruction of the Bowel and Mechanical Displacement of Organs.* By JOHN MARSHALL, Esq.

Mrs. B., æt. 41, the wife of a corn dealer, residing at B——, in Oxfordshire, was a rather tall well-formed woman of fair complexion, good intelligence and habitually benevolent to the poor.

In the year 1844 I first attended her in childbirth. The labor was natural, and she soon recovered. My father had previously been her medical friend, and was with her at the birth of five children; a fortnight after the birth of the fifth child, in December, 1842, she was affected with hæmatemesis, vomiting a wash-hand basin full of blood. During the succeeding forty-eight hours she lay in a state of total unconsciousness, pupils greatly dilated, and the pulse scarcely perceptible. The vomiting did not return, and she slowly recovered, but her countenance ever after was sallow.

In the autumn of 1845 I found her complaining of frequent sickness, with pain at the epigastrium, and in the left groin. On examining the abdomen, I discovered a hard tumour in the left iliac fossa, which moved freely across the abdomen, as she turned from side to side. The size and shape of this tumor were very like an ordinary placenta, one edge thicker than the other; the thickest edge much harder, and imparting to the fingers the feeling of its being very heavy. She stated that she had felt this substance for some months, and whenever she turned in bed it always moved, and caused nausea, but gave her no pain when she was quiet, nor was it tender to the touch. She suffered much from flatulence, and pain between the shoulders shooting through to the left breast. The catamenia had not appeared for three months, and she believed she was pregnant. The bowels were torpid, and she was obliged frequently to take her usual aperient of blue pill and colocynth. The nausea increased, and the stomach rejected everything; large quantities of green ropy mucus, occasionally with blood, were thrown up, all of which was carefully examined, without detecting anything which would throw light on the case. Numerous remedies were employed to allay her distressing sickness, but were equally unavailing, and the emaciation and exhaustion became so great, that her death was daily expected. One teaspoonful of pale brandy was now given every hour, and not an atom or drop of anything beside. From this time the vomiting ceased. After continuing this for two days a little food was allowed, and she gradually gained strength and flesh, and in four months was able to walk two miles, and looked almost as well as usual.

During this illness, Dr. Cowan, of Reading, visited my patient; he was greatly interested in her case, and expressed himself to be as much puzzled as I was respecting the nature of the tumor, which he compared to a cannon ball rolling across the abdomen.

Nearly five years elapsed before any serious symptom returned. The pain between the shoulders was often complained of, and there was occasional sickness. Much difficulty was experienced in keeping the bowels in order; the secretions were generally healthy in color, and formed. Occasionally the face and limbs became œdematous, but an aperient pill, with a little gin in gruel taken at bedtime, would always remove these symptoms in two or three days. The catamenia never returned after the illness of 1845.

On the 8th of October, 1850, I again saw her in consultation with Mr. Corsillis, of B——. From him I learned that she had been ill three weeks, and all her old symptoms had returned. Incessant vomiting with pain between the shoulders, had reduced her to a state of great weakness and emaciation. A few hours after my visit, severe spasmodic pain came on in the bowels, and quickly terminated her sufferings.

*Post-Mortem.*—Eighteen hours after death, with the assistance of Mr. Corsillis, I examined the body. The thoracic viscera were healthy and free from adhesions. On opening the abdomen the stomach was found drawn down to the pubis, and in its form resembled a champaign bottle. The pyloric end lay beneath the arch of the pubis and the duodenum under a portion of the sigmoid flexure of the colon, from which it was traced to the pancreas, which was drawn down considerably out of its normal position. The liver was large and paler than natural. The gall-bladder distended with bile. The spleen and pancreas were healthy, but small. The kidneys rather hypertrophied. The bladder small. The uterus and ovaria healthy. The intestines were of very small calibre. The cœcum and colon resembled the small intestines, the bands and sacciolated appearance being scarcely discernible. The jejunum and ileum contained a little ropy mucus, and there was some fœcal matter in the rectum. No ulceration was apparent throughout the whole length of the intestinal canal, nor was there found the slightest peritoneal attachment or appearance of inflammation within the cavity of the abdomen.

Having tied the œsophageal end of the stomach and the duodenum, I removed these organs. The stomach contained about a pint of semi-fluid matter, and felt very like the crop of a fowl; the duodenum resembled a large sausage stuffed with lead. On cutting into the stomach I found it partially filled with some gruel-like fluid, and in the lower half—which evidently constituted the tumor during life—an immense number of pins, of a purple black color, not corroded, varied in size, all bent or broken, many very pointed. The pyloric half of the stomach presented a remarkably thickened condition of the villous coat, being highly vascular, and raised in rugous elevations like the stomach of an ox. The muscular coat also was greatly hypertrophied. The weight of the pins contained in the stomach was *nine ounces.* An incision made into the duodenum *displayed a mass of pins very tightly packed, of various shapes, similar to those found in the stomach, and wholly obstructing the tube.* These weighed a *pound,* as nearly as I could ascertain without removing them.

Her husband could scarcely believe the truth of what he saw, when we showed him the contents of the stomach, for he affirmed that he had never seen his wife put pins into her mouth. A son, 17 years of age, said that he had often observed his mother biting pins, and believed that she swallowed them. She took them out of her thimble with her tongue, having previously bent the head and point together. When his mother corrected him for any bad habit, he would say—why do you eat pins? This reply always silenced her. He stated that the servants when shaking the carpets, frequently remarked on the number of bent pins they found.

Mrs. B—— had a keen appetite, and would always partake of any food she fancied, however improper or indigestible. I have known her to eat cold boiled pork when unable to raise herself in bed, having four days previously vomited a large quantity of blood. Of her early history I can learn but little. A sister informs me that when a child she was fond of eating starch and slate pencil—and she remembers her biting pins. When seventeen years of age she vomited blood, and remained for some years an invalid, until sent for change of air to Bath.—*Medico-Chirurgical Transactions.*

